# Age structure changes indicate direct and indirect population impacts in illegally harvested black rhino

**DOI:** 10.1371/journal.pone.0236790

**Published:** 2020-07-29

**Authors:** Nikki le Roex, Sam M. Ferreira

**Affiliations:** 1 Scientific Services, South African National Parks, Skukuza, South Africa; 2 Institute for Communities and Wildlife in Africa (iCWild), Department of Biological Sciences, University of Cape Town, Cape Town, South Africa; Universita degli Studi di Sassari, ITALY

## Abstract

Overharvesting affects the size and growth of wildlife populations and can impact population trajectories. Overharvesting can also severely alter population structure and may result in changes in spatial organisation, social dynamics and recruitment. Understanding the relationship between overharvesting and population growth is therefore crucial for the recovery of exploited species. The black rhinoceros (*Diceros bicornis*; black rhino) is a long-lived megaherbivore native to sub-Saharan Africa, listed as Critically Endangered on the IUCN Red List of Threatened Species. Since 2009, the targeted illegal killing of rhino for their horns has escalated dramatically in South Africa. Given their slow life trajectories, spatial structure and social dynamics, black rhino may be susceptible to both direct and indirect impacts of overharvesting. Our study compared black rhino demography before and during extensive poaching to understand the impact of illegal killing. The population exhibited significant changes in age structure after four years of heavy poaching; these changes were primarily explained by a decrease in the proportion of calves over time. Population projections incorporating both direct poaching removals and decreased fecundity/recruitment were most similar to the observed demographic profile in 2018, suggesting that indirect impacts are also contributing to the observed population trajectory. These indirect impacts are likely a result of decreased density, through processes such as reduced mate-finding, population disturbance and/or increased calf predation. This study illustrates the combined effect of direct and indirect impacts on an endangered species, providing a more comprehensive approach by which to evaluate exploited populations.

## Introduction

Overharvesting of wildlife populations is a central concern of conservation biology. Overharvesting can occur as a result of poorly-regulated legal harvest (e.g. sport hunting; [1]), subsistence removals (e.g. bush-meat snaring; [2]), population control strategies (e.g. culling; [3]) and illegal killing for profit (e.g. ivory poaching; [4]). Large mammals are often the targets of such practices, but the extent and consequences of such activities can be difficult to assess, particularly for illegal activities where information must be gathered indirectly. Overharvesting affects both population size and growth as a result of the direct mortalities suffered, and can have profound consequences on population trajectories [5]. Continued removals over time, as is often seen with illegal killing, can also profoundly alter the structure of a population [6]. Large mammal populations exhibit defined age and sex structures, often with class-specific survival rates [7]; as a result, changes in population structure can severely alter temporal dynamics and the population-level response to stochastic environmental variation [8]. Processes that also alter the structure of populations can therefore have severe consequences on the growth and trajectories of endangered species.

In addition to the direct mortalities of overharvesting, changes in population structure can also indirectly affect fecundity, recruitment and reproduction [8,9]. Overharvesting can induce behavioural changes that may carry fitness costs as a result of decreased energy intake or increased energy expenditure [10]. Human-induced selection–intentional or unintentional–has the potential to cause rapid phenotypic and evolutionary change in exploited populations that may reduce viability [11,12]. Overharvest may also cause changes in spatial organisation [13,14], social dynamics/interactions [15] and recruitment [16,17], which can significantly affect population growth [9]. Understanding the relationship between overharvesting and the indirect effects on population growth and viability is therefore crucial for the recovery of exploited species.

The black rhinoceros (*Diceros bicornis*; black rhino) is a long-lived megaherbivore native to sub-Saharan Africa. Once widespread across the continent, the population declined by 97% during the 1900’s as a result of overharvesting, and although subsequent conservation initiatives increased numbers to the current population of 5,250 animals, the black rhino remains Critically Endangered on the IUCN Red List of Threatened Species [18]. The Kruger National Park (Kruger) hosts one of the largest black rhino populations in South Africa, which grew from 81 re-introduced founders, beginning in 1971, to an estimated 627 (95% CI: 588–666) animals in 2009 [19]. Analysis of the population vital rates in 2009 showed 6.75% growth per annum, high adult and calf survival and an average inter-calving interval (ICI) of 2.45 years [19]. In 2009, however, the targeted illegal killing of rhino (poaching) for their horns began in earnest within Kruger. Rhino horn is one of the most sought-after commodities in the illegal wildlife trade and can fetch prices in excess of US $35,000 per kilogram [20]. The demand originates primarily in Asia, with rhino horn used as a traditional medicine to treat ailments such as fevers, headaches and cancer [21]. More recently, demand has extended to include rhino horn as a status symbol among the wealthy and a financial investment relying on species extinction for value increase [21].

Black rhinos are elusive mammals and are considered an asocial species [22], but increasing evidence suggests that their social dynamics and interactions are complex [23] and potentially mediate population vital rates [24]. Black rhino exhibit a polygynous mating system [25,26], structured around well-defined spatial organisation. Males are territorial and compete for social and breeding dominance from 8–10 years of age [27], and their territories typically overlap the home ranges of several adult females [28]. Female black rhino usually exhibit overlapping home ranges [28], and may engage in female-based philopatry by sharing part of their home ranges with adult female offspring. Black rhino have a gestation time of 15 months, with age at first calving around seven years [22,25]. Rhino calves are dependent on their mothers for milk for the first year after birth and protection for the first two years, as they are vulnerable to predation primarily by spotted hyenas (*Crocuta crocuta*) and possibly lions (*Panthera leo*; [29]). Reproductive senescence has been suggested to occur between 30–35 years of age, with a similar lifespan in the wild [22,30].

In recent years, the proportion of black rhino killed in Kruger relative to their population size has increased, raising concerns regarding the impact of poaching on the stability and viability of the population. Given their slow life-trajectories, spatial structure and potentially complex social dynamics, black rhino may be susceptible to both direct and indirect effects of overharvesting. Our study compared the population demography of black rhino in Kruger before and during extensive poaching to infer the direct and indirect impacts of illegal killing on the population stability and trajectory. More specifically, we: (i) compared pre-poaching age structure with the annual population structure seen over five years after the poaching upsurge, (ii) determined operational sex ratios before and during extensive poaching, and (iii) investigated calf proportion changes between the pre-poaching and poaching period. Finally, we simulated population growth under different poaching and fecundity scenarios and compared the outputs to the current population size and structure to determine the possible contribution of indirect poaching effects to population demography.

## Materials and methods

### Study site

**Fig 1:**
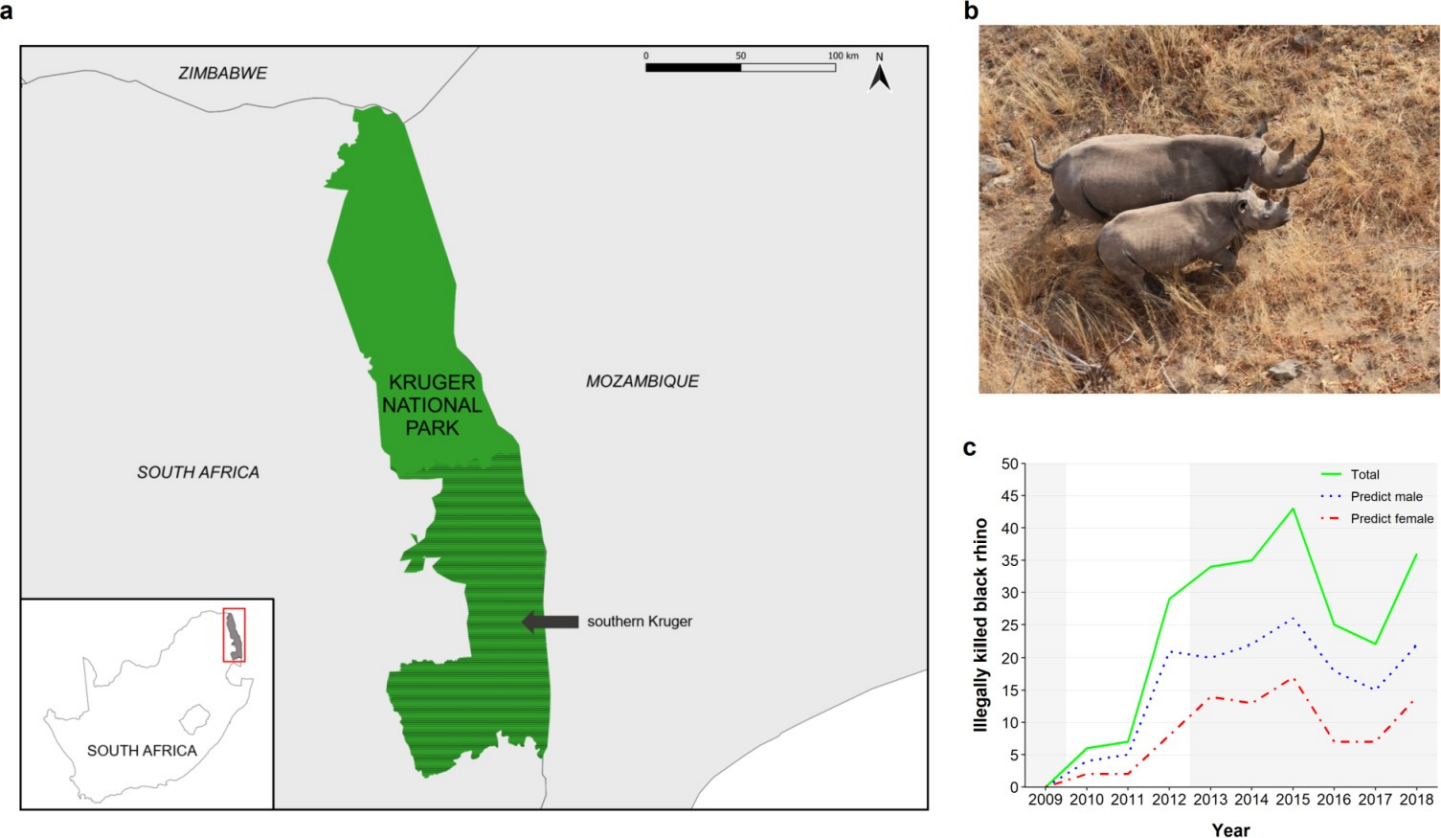
(a) The Kruger National Park, South Africa. (b) Black rhino cow and calf as seen during aerial surveys. (c) Number of illegally killed black rhino from 2009–2018. Dotted lines indicate the predicted sex composition of annual poaching incidences, based on the ratio of known-sex carcasses across all years. Shaded grey indicates the years of population data used for this study.

The Kruger National Park (24°0′41″ S, 31°29′7″ E) is South Africa’s largest protected area, encompassing more than 19,500 km^2^ (Fig 1A). While mean temperature and rainfall varies considerably along a north-south gradient, summers are typically hot and wet and winters are warm and dry. Black rhino historically occurred throughout this semi-arid savanna ecosystem prior to their local extinction in 1936 following an extended period of targeted hunting [31]. Black rhino re-introductions occurred within the southern region (south of the Olifants River; Fig 1A) of the park [32], with 81 animals re-introduced from Zimbabwe and KwaZulu Natal, South Africa, in the 1970’s and 1980’s. Black rhino have remained primarily within this area to date. Landscape types within the park are comprised of mixed species woodland on granite and gneiss deposits in the west, and wooded savanna on nutrient-rich basalts in the east [33].

### Data

We used observation data of black rhinos collected during aerial surveys in southern Kruger from 2009 to 2018 (SANParks; Fig 1B). Ethics approval was not required as no animals were handled for this study; the data utilised was collected non-invasively by South African National Parks (SANParks) as per the organisation’s Wildlife Management Policy. Surveys occurred at the end of the dry season each year, eliminating any effects of month or season on age class frequencies. These observations included age class classification for all individuals and sex classification for adult animals. Black rhinos were aged by standard age classes A-F for rhinos (A: 0–3 months, B: 3–12 months, C: 1–2 years, D: 2–3.5 years, E: 3.5–7 years, and F: 7 years and older; [34]). As we were interested in any changes in functional composition of the black rhino population for this study, we did not require the fine-scale resolution of every age class. In order to increase observation numbers per category, remove possible uncertainty in A and B class assignments and make the data more functionally relevant, we collapsed the age classes into the following categories: ‘calf’ (0–2 years; A, B and C classes), ‘sub-adult’ (2–7 years; D and E classes) and ‘adult’ (7+ years; F class). Poaching of black rhino in Kruger began in 2010, with 6–43 animals killed annually in subsequent years (SANParks; Fig 1C). Annual mortality data included cause and estimated date of death of each rhino carcass found, and sex and age group were recorded when known. Only rhino deaths recorded as poached were included in the analyses as natural deaths were assumed to be reflected in annual survival rates.

### Temporal demographic comparison

We compared age category frequencies in each year from 2013–2018 (poaching) to 2009 (pre-poaching). For each comparison, we used chi-squared tests to compare the frequency of individuals in each age category in the poaching year to the frequency obtained pre-poaching. Following the method of Jones et al. (2018), we then calculated the standard residuals (SR) between the observed (O) and expected (E) frequencies for each age category between each poaching year and the pre-poaching frequencies, using the formula SR = (O-E)/√E. Negative and positive standard residuals indicate observed frequencies that were lower and higher than expected, respectively. To test for evidence of sex-bias in the poaching of black rhino, we calculated the ratio of adult females to adult males (operational sex ratio) for all years and compared the frequency obtained in each poaching year to the pre-poaching ratio using chi-squared tests. Finally, we tested whether the ratio of adult females per dependent calf had changed over the study period. We calculated the number of adult females per dependent calf (‘calf’ category; 0–2 years) in the population for each year. In order to estimate the total number of adult females per year, given variable proportions of unknown sex adults per year, we assigned sex to unknown sex adults based on a 1:1 ratio. We compared this ratio in each poaching year with pre-poaching ratios using chi-squared tests. All analyses were performed in R v3.5.3 [35].

### Population projection

In order to visualise the possible black rhino population trajectories over this time period, we used age-based population projection models to simulate population growth from 2009 to 2018 under three different poaching scenarios, using different age and sex ratios of animals killed and, in some scenarios, adjusting for the likely death of dependent calves that were never found (Table 1). Rhino calves left without the protection of their mothers are quickly killed by predators and are consumed in their entirety; rhino calf carcasses are never found in Kruger but clearly occur (pers comm.). For all poaching scenarios, pre-poaching population size and survival rates from Ferreira et al. (2011) were used in the population projections. These were 0.99 for ages 0–1 year (males and females), 0.94 for 2–4 years (males), 0.99 for 2–4 years (females), 0.64 for 5–6 years (males), 0.82 for 5–6 years (female) and 0.99 for 7+ years (males and females). Male and female projection matrices were compiled and run separately, assuming a 1:1 birth sex ratio.

**Table 1:** Poaching scenarios tested in the population projections from 2009–2018. The annual number of black rhino poached was retained as documented, but age and sex ratios and the removal of dependent calves varied between scenarios.

Scenario	Female: Male	Adult: Sub-adult	Predicted calves removed
Recorded	1:1.6	1:0	No
No sex bias + calves	1:1	1:0.33	Yes
No sex/age bias + calves	1:1	1:1	Yes

In scenario 1, annual adjustments were made to include recorded poaching incidences according to the ratio of known-sex carcasses in the SANParks rhino carcass database (‘recorded’). We calculated the sex ratio of known-sex poached carcasses across all years from 2009 to 2018. We then assigned sex to the unknown carcasses each year based on that ratio and added the expected males and females to the known males and females, respectively, to obtain predicted annual totals for each sex (Fig 1C). In scenario 2, annual adjustments were made to include recorded poaching incidences, assigning an equal adult sex ratio to unknown sex individuals and removing the predicted dependent calves killed but never found (‘no sex bias + calves’). In scenario 3, annual adjustments were made to include recorded poaching incidences, assigning equal sex and adult:sub-adult ratios, and removing the predicted dependent calves killed but never found (‘no sex/age bias + calves’).

Age group of illegally killed black rhinos is difficult to assign for the sub-adult and adult categories, in part due to the absence of horns on carcasses and the speed at which tissue is removed from bone; more than 92% of carcasses with recorded ages were categorised as adults (SANParks). Thus for the recorded poaching scenario, we disregarded age group and ran the population projection with all carcasses as adults. For the latter two scenarios which included dependent calves that were likely killed but never found, we used the number of illegally killed adult females per year in each scenario multiplied by the annual proportion of adult females expected to have a dependent calf at foot. For the annual poaching deductions, individuals were removed from the projection vectors by random sampling across the applicable age years. An annual birth rate of 0.33 was used, as a conservative estimate based on the predicted ICI of 2.45 years calculated in 2009 [19]. We compared the predicted population size, age structure, operational sex ratio and dependent calf to adult female ratio under the three different poaching scenarios with the corresponding metrics observed in 2018 to explore the extent to which direct poaching mortalities accounted for the population changes seen.

Finally, to explore the extent of indirect effects, we repeated the population projections under the same poaching scenarios and initial demographic parameters, but decreased the reproductive rate of females. We used ICIs of 4, 5 and 6 years as our proxies for indirect effects; this would encompass decreased calf survival (pre-natal or post-natal) as well as increased time between successive pregnancies. Inter-calving intervals are influenced by many social, spatial and demographic factors likely impacted as a result of overharvesting and therefore are a suitable proxy for combined indirect effects. We compared the resulting trends in predicted population size, age structure, dependent calf to adult female ratio, and operational sex ratio to the corresponding metrics observed in 2018. Population projections were performed in R v3.5.3 [35].

## Results

### Temporal demographic comparison

**Fig 2:**
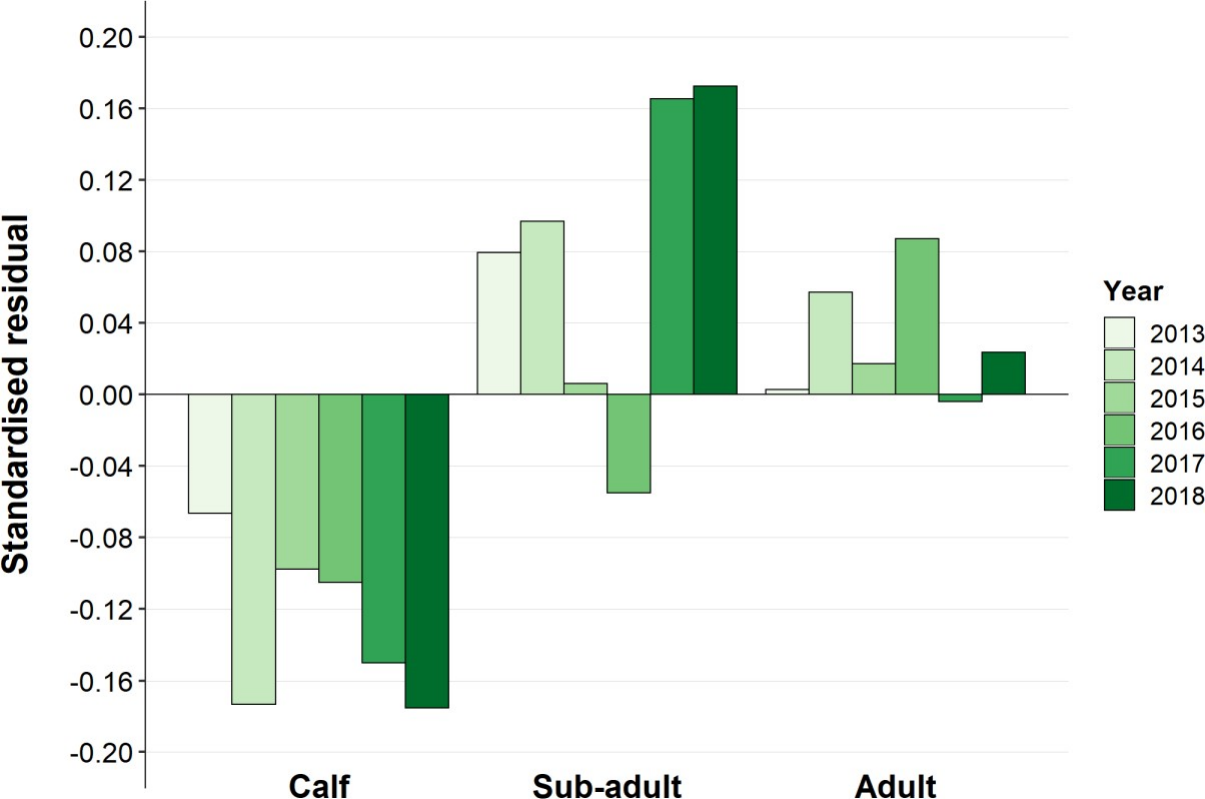
Standardised residuals from chi-squared tests comparing age group frequencies from 2013 to 2018 (poaching) to those observed in 2009 (pre-poaching).

The number of black rhino observations in southern Kruger ranged from 122–233 per year. The age category structure of the black rhino population in the pre-poaching period was 22.1% dependent calf, 13.6% sub-adult and 64.3% adult (Table 2). Comparison of the age group frequencies between the pre-poaching and poaching years showed no significant difference until 2017. Both 2017 and 2018 age structures were significantly different to those observed in 2009 (Table 2). The population showed an increase in the proportion of sub-adults and a decrease in the proportion of dependent calves with time, and a marginal increase in the proportion of adults (Fig 2).

**Table 2:** Black rhino age group frequencies per year. Chi-squared statistics (χ^2^) and *p*-values relate each subsequent year to the 2009 age group frequencies.

Year	Total	Calf	Sub-adult	Adult	Sex (adult)	χ^2^	*P* value
2009	154	34	21	99	F:53, M:46	-	-
2013	169	32	28	109	F:40, M:44, U:25	1.8225	0.402
2014	122	17	21	84	F:21, M:27, U:36	5.2049	0.074
2015	166	29	23	109	F:33, M:42, U:34	1.5474	0.4613
2016	181	31	21	129	F:65, M:58, U:6	3.9324	0.14
2017	233	35	46	149	F:79, M:63, U:7	11.74	0.0028*
2018	195	27	39	129	F:69, M:59, U:1	11.882	0.0026*

* *P* < 0.05

**Fig 3:**
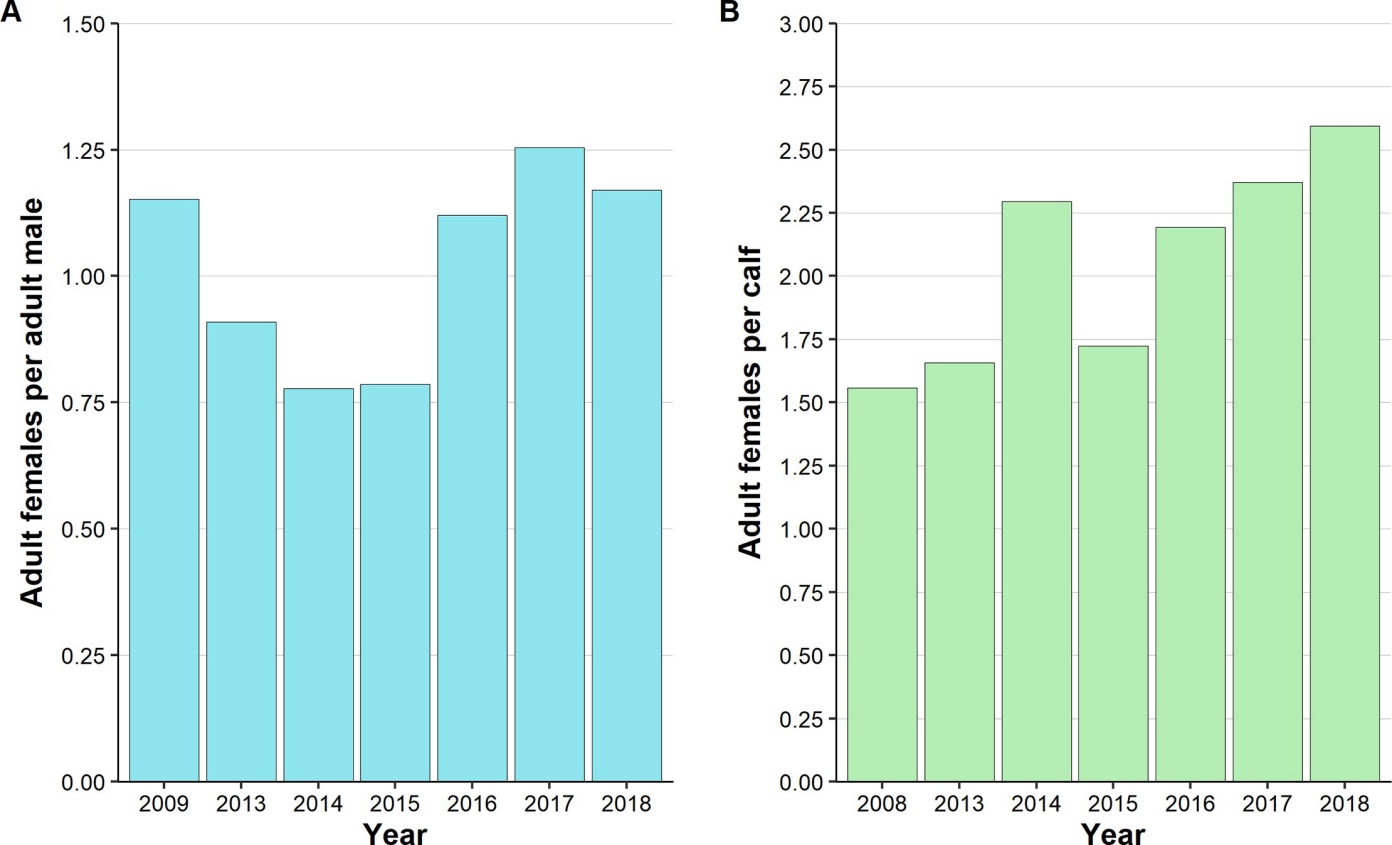
Trends in (a) operational sex ratio, and (b) adult female per dependent calf ratio for black rhino in 2009 (pre-poaching) and 2013–2018 (poaching). Note that the scale of the axes differ between graphs.

The operational sex ratio of the black rhino population showed no significant differences between pre-poaching and poaching years (range = 0.78–1.25; Chi-squared: 0.007–2.741, all p > 0.05; S1 Table). There was, however, a decreasing trend in the proportion of adult females from 2013–2015, followed by an increase in females in 2016 that remained fairly constant until 2018 (Fig 3A). The number of adult females per calf was significantly higher in both 2017 and 2018 compared to 2009 (Table 3). Annual ratios showed a fairly consistent increasing trend over time (Fig 3B).

**Table 3:** Annual ratios of adult female black rhinos per calf. Chi-squared statistics (χ^2^) and *p*-values compare subsequent years to 2009.

Year	Adult females per calf	χ^2^	*P* value
2009	1.56	-	-
2013	1.66	0.0734	0.7865
2014	2.29	1.7899	0.1809
2015	1.72	0.1866	0.6657
2016	2.19	2.5088	0.1132
2017	2.37	4.3976	0.0360*
2018	2.59	5.1524	0.0232*

* *P* < 0.05

### Population projection

**Fig 4:**
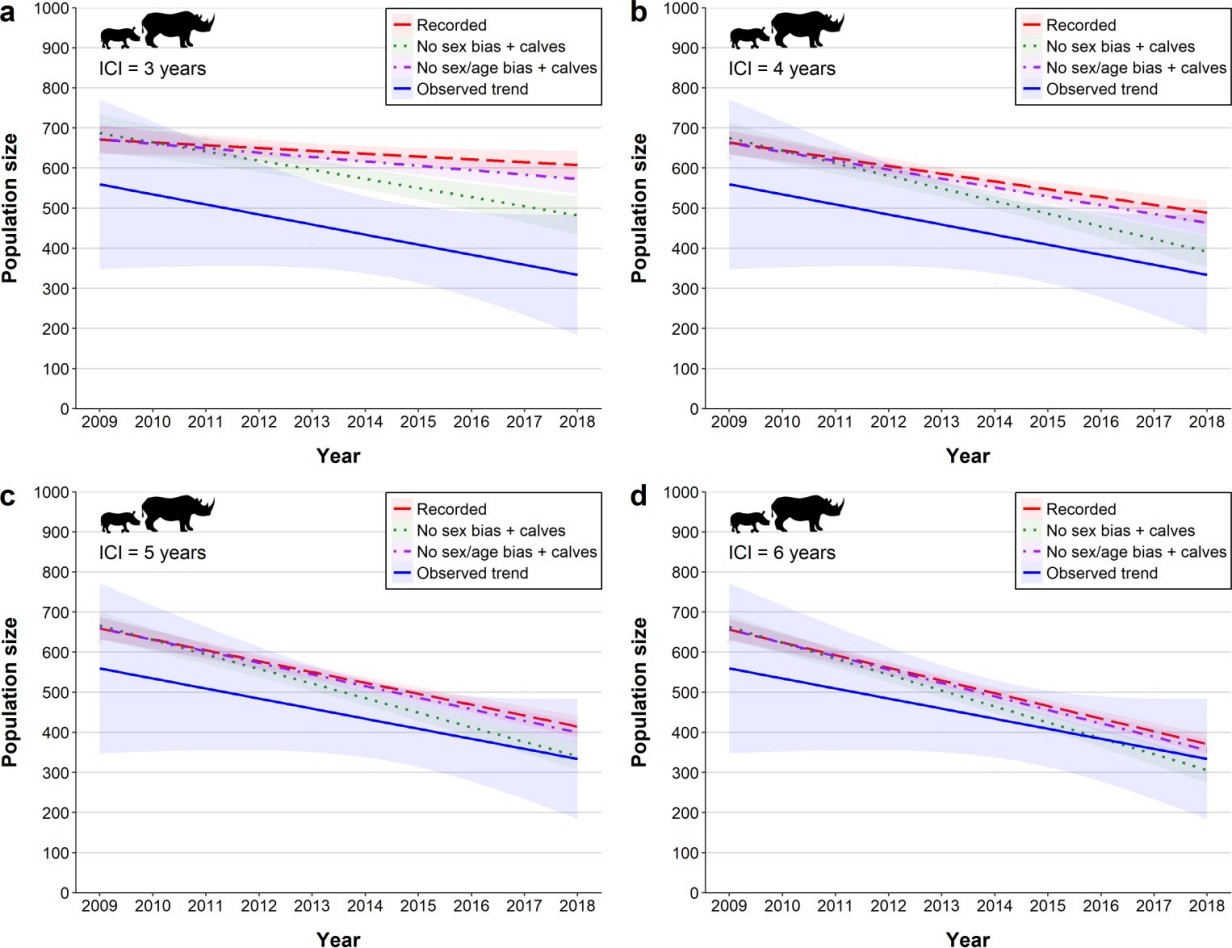
Population size projections under ‘recorded poaching’, ‘no sex bias + calves’, ‘no sex/age bias + calves’ poaching scenarios with inter-calving intervals of (a) 3, (b) 4, (c) 5 and (d) 6 years compared to the observed census trend for black rhino from 2009–2018. Shaded areas represent 95% confidence intervals of the estimated trends.

The 2018 population estimate from the annual rhino survey was 291 (95% CI: 151–441) black rhinos (SANParks internal report). Simulating the direct poaching mortalities under the three scenarios using the pre-poaching demographic parameters resulted in population size projections from 461 (no sex bias + calves) to 589 animals (recorded poaching; Fig 4A). Age structure projections for all poaching scenarios were significantly different to that observed in 2018 (Chi-squared = 48.75–146.96, all p <0.01; S2 Table). More specifically, the observed proportions of calves and sub-adults were considerably lower than any of the simulated calf and sub-adult proportions, and the proportion of observed adults was higher. Similarly, both the operational sex ratio (range = 1.48–2.97, Chi-squared = 4.52–46.19, all p <0.05; S3 Table) and dependent calf to adult female ratio (range = 1.56–2.59, Chi-squared = 5.33–17.18, all p <0.05; S4 Table) were significantly different to those observed in 2018 (dependent calf: adult female = 2.59; operational sex ratio = 1.17) for all poaching scenarios.

When including the proxy for indirect poaching effects, increased ICIs of five and six years produced the most similar population size trends to those observed in 2018 (observed: 151–441; ICI 5: 334–408 animals; ICI 6: 303–367 animals; Fig 4). Similar age structures were simulated under the ‘no sex/age bias + calves’ scenario for ICI 5 and 6 (ICI 5: chi-squared = 1.23, p = 0.5397; ICI 6: chi-squared = 0.71, p = 0.7026; S1A Fig) to those observed in 2018; all other simulations produced significantly different age group proportions (S2 Table). Simulated operational sex ratios converged on those observed in 2018 under the ‘no sex/age bias + calves’ scenario with ICI’s from 4–6 (ICI 4: chi-squared = 3.264, p = 0.07082; ICI 5: chi-squared = 3.3021, p = 0.06919; ICI 6: chi-squared = 2.9854, p = 0.08402; S1B Fig); all other simulations were significantly different (S3 Table). Dependent calf to adult female ratios in all poaching scenarios were similar to those observed in 2018 for ICI 4, ICI 5 & ICI 6 (S4 Table).

## Discussion

The Kruger black rhino population displayed a significantly different age structure to the structure seen in the pre-poaching period after only four years of heavy poaching. These changes were primarily explained by a decrease in the proportion of dependent calves and an increase in the proportion of sub-adults over time. The ratio of adult females per dependent calf in 2018 was almost double that seen in the pre-poaching period, despite a similar proportion of adults in the population. Population projections incorporating both direct removals and indirect effects (decreased fecundity/recruitment) were most similar to the observed demographic profile in 2018 when poaching was simulated with no sex or age bias, the likely dependent calves of poached females were removed and inter-calving interval was increased to 5 or 6 years.

In our study, both population age structure and the number of adult females per dependent calf changed significantly in 2017 and 2018 compared to the equivalent pre-poaching values in 2009. This suggests that the effect of the direct removals took some years (or some proportion of population removals) to manifest in a detectable change in these metrics. Operational sex ratios did not change significantly, indicating a lack of intentional or unintentional sex bias in poached black rhinos. As there was no survey data prior to the onset of poaching in 2009, neither stochastic population variation nor sampling variation could be definitively estimated in this system. However, annual stochastic variation in population composition in a long-lived mammal with a long gestation period such as black rhino is expected to be low. Furthermore, by combining traditional rhino age classes into functional classes that spanned multiple years, we reduced potential sampling variation across surveys. While overharvesting is generally expected to affect both ends of a population age distribution, in our study it appeared to disproportionately impact the dependent calf age category. This is unlikely purely the result of direct removals, even if the dependent calves that die after their mothers have been killed (and whose carcasses are never found) are considered. For the calf age category to be more affected than the adult category to which their mothers belong, additional factors (indirect effects) must be impacting this black rhino population. A decrease in the proportion of dependent calves following the removal of older individuals has also been seen in elephant populations subjected to poaching pressure [6], and is considered to reflect suppressed recruitment as a function of indirect impacts on breeding and/or calf survival.

Demographic Allee effects [36] encompass a range of mechanisms under which a population experiences decreased growth as a result of decreased population size [37]. Cooperative interaction mechanisms include any process where fitness is reduced by reducing conspecific interactions, such as fewer mating opportunities [38,39] or increased mortality due to insufficient defence strategies by smaller groups [40]. Mate-finding, in particular, has been the focus of many studies; if a proportion of individuals are not mated to their full potential, fewer offspring per individual over time can lead to substantial declines in population growth [39]. Given the large expanse of southern Kruger and the uneven distribution of black rhinos across this landscape, it is possible that interactions between individuals are spatially or temporally reduced. Both male and female black rhino maintain well-defined home ranges and are slow to disperse into or recolonize vacant areas [23,41]. The regular removal of individuals by poaching is therefore likely to increase the distances between animals, and/or decrease home range overlap, potentially for many months or years. Linklater and Hutcheson [23] showed that female black rhino did not change their spatial organisation when a neighbouring/overlapping male was harvested from the population, while males decreased their range when a neighbouring female was removed. While the behavioural mechanisms that govern receptivity and mating are poorly understood, the spatial organisation of black rhino is likely an important component of their breeding system and reduced interactions between the sexes at low population density may delay mating and thus increase inter-calving intervals. Hrabar and Toit [42] found that maternal success increased with population density of black rhino in Pilanesberg, South Africa, supporting the importance of cooperative mechanisms within black rhino populations.

Overharvesting of individuals can disrupt the established social dynamics of a population; these perturbation effects are particularly likely to impact populations that exhibit highly structured socio-spatial systems [43]. Increasing the rate of social re-organisation in a population can increase the frequency and intensity of encounters between new individuals [16]. For example, increased male turnover has been demonstrated to increase infanticide thereby decreasing recruitment in large carnivores such as leopards (*Panthera pardus*; [44]), lions (*Panthera leo*; [1]) and brown bears (*Ursus arctos*; [45]). Increased male turnover may also increase natural deaths from territorial fights [44] and decrease adult survival. Furthermore, females may be reluctant to mate when dominant male tenure has been disrupted and a new dominant male has not yet established himself. Frequent turn-over of males as a result of continued poaching in the same area may therefore inadvertently slow breeding rates until social stability is regained. In black rhinos, intra-specific fighting is frequent and often results in injury and/or death [29]; avoiding conflict until the local male hierarchy has stabilised would likely increase survival of both the female and her future offspring.

The change in dependent calf recruitment may, however, also be influenced by increased predation. While adult black rhino are seldom at risk, calves and sub-adults are vulnerable to predation by large predators [29,46]. Observations of black rhino calves with mutilated pinnae and/or missing tails in the Hluhluwe-iMfolozi Complex, South Africa, were attributed to hyena attacks, and subsequent calf disappearance suggested that predation significantly contributed to calf mortality [29]. These findings supported early observations of black rhino calf vulnerability to hyena predation [46]. The hyena population in southern Kruger increased from 3,667 (95% CI: 3443–3891) in 2008 to 7,339 (95% CI: 6998–7680) animals in 2015 (SANParks internal report), and while the drivers of this population growth are unknown, the increase may be related to food availability. The number of rhino carcasses (both black and white) available as a result of poaching increased from 50 to 826 per year from 2009 to 2015 (SANParks, unpubl. data), providing a dramatic increase in easily accessible meat for scavengers annually. Numerous observations of black rhino calves and adults without one or both ears in southern Kruger further supports the potential role of hyena predation on black rhino calf mortality. Lion attacks on black rhino have been recorded on occasion [47,48], but are generally considered infrequent and are therefore unlikely to be a major threat to dependent calf recruitment. Increased predation may also be linked with reduced black rhino density, as single adult black rhino are likely to be less of a deterrent to hyenas and thus a more appealing target for calf predation. Increased calf mortality by hyena predation can, to some degree, also be considered an indirect overharvesting impact, if the increase in rhino carcasses drove the population explosion.

In conclusion, while there has been much focus on the prevention of rhino poaching, little research has been directed towards understanding or mitigating the potentially devastating effects of such frequent removals on the reproductive dynamics of black rhino. The impact of reduced density is considered to be the proximate cause of drastically reduced breeding rate, reproductive malfunction and ultimately, the wild population demise of the Sumatran rhino (*Dicerorhinus sumatrensis*; [49]), further supporting the role of Allee effects as a primary driver of rhino population dynamics. Future work should attempt to untangle the drivers of recruitment declines in black rhino so that both direct and indirect overharvesting impacts may be addressed, and ultimately ensure that black rhino populations under such extreme pressures do not follow a similar trajectory.

## Supporting information

S1 Fig (a) Age group proportions of black rhino predicted in 2018 under ‘no sex/age bias + calves’ for ICI 5 and 6 compared to those observed in 2018. (b) Proportions of females and males predicted in 2018 under ‘no sex/age bias + calves’ for ICI 4, 5 and 6 compared to those observed in 2018.
(TIF)

S1 Table Operational sex ratios of black rhino observed in 2009 and 2013–2018. Chi-squared statistics compare subsequent years to 2009.
(DOCX)

S2 Table Projected black rhino age group proportions in 2018 under different poaching and fecundity scenarios. Chi-squared statistics compare projected to observed 2018 proportions.
(DOCX)

S3 Table Projected operational sex ratios of black rhino in 2018 under different poaching and fecundity scenarios. Chi-squared statistics compare projected to observed 2018 ratios.
(DOCX)

S4 Table Projected ratios of adult female black rhino per dependent calf in 2018 under different poaching and fecundity scenarios. Chi-squared statistics compare projected to observed 2018 ratios.
(DOCX)

## References

[1] Packer C, Kosmala M, Cooley HS, Brink H, Pintea L, Garshelis D, et al Sport hunting, predator control and conservation of large carnivores. PLOS ONE. 2009;4: e5941 10.1371/journal.pone.0005941 19536277 PMC2691955

[2] Rogan MS, Lindsey PA, Tambling CJ, Golabek KA, Chase MJ, Collins K, et al Illegal bushmeat hunters compete with predators and threaten wild herbivore populations in a global tourism hotspot. Biological Conservation. 2017;210: 233–242. 10.1016/j.biocon.2017.04.020

[3] Rushton SP, Shirley MDF, Macdonald DW, Reynolds JC. Effects of culling fox populations at the landscape scale: A spatially explicit population modeling approach. Journal of Wildlife Management. 2006;70: 1102–1110. 10.2193/0022-541X(2006)70[1102:EOCFPA]2.0.CO;2

[4] Wittemyer G, Northrup JM, Blanc J, Douglas-Hamilton I, Omondi P, Burnham KP. Illegal killing for ivory drives global decline in African elephants. PNAS. 2014;111: 13117–13121. 10.1073/pnas.1403984111 25136107 PMC4246956

[5] Treves A, Naughton-Treves L. Evaluating lethal control in the management of human-wildlife conflict In: Woodroffe R, Thirgood S, Rabinowitz A, editors. People and Wildlife, Conflict or Co-existence? Cambridge, United Kingdom: Cambridge University Press; 2005 10.1016/j.jtbi.2005.03.003

[6] Jones T, Cusack JJ, Pozo RA, Smit J, Mkuburo L, Baran P, et al Age structure as an indicator of poaching pressure: Insights from rapid assessments of elephant populations across space and time. Ecological Indicators. 2018;88: 115–125. 10.1016/j.ecolind.2018.01.030

[7] Gaillard JM, Festa-Bianchet M, Yoccoz NG. Population dynamics of large herbivores: variable recruitment with constant adult survival. Trends in Ecology & Evolution. 1998;13: 58–63. 21238201 10.1016/s0169-5347(97)01237-8

[8] Milner JM, Nilsen EB, Andreassen HP. Demographic side effects of selective hunting in ungulates and carnivores. Conservation Biology. 2007;21: 36–47. 10.1111/j.1523-1739.2006.00591.x 17298509

[9] Frank SC, Ordiz A, Gosselin J, Hertel A, Kindberg J, Leclerc M, et al Indirect effects of bear hunting: a review from Scandinavia. Ursus. 2017;28: 150–164. 10.2192/URSU-D-16-00028.1

[10] Lima SL, Dill LM. Behavioral decisions made under the risk of predation: a review and prospectus. Canadian Journal of Zoology. 1990;68: 619–640. 10.1139/z90-092

[11] Allendorf FW, Hard JJ. Human-induced evolution caused by unnatural selection through harvest of wild animals. PNAS. 2009;106: 9987–9994. 10.1073/pnas.0901069106 19528656 PMC2702803

[12] Hendry AP, Farrugia TJ, Kinnison MT. Human influences on rates of phenotypic change in wild animal populations. Molecular Ecology. 2008;17: 20–29. 10.1111/j.1365-294X.2007.03428.x 18173498

[13] Leclerc M, Frank SC, Zedrosser A, Swenson JE, Pelletier F. Hunting promotes spatial reorganization and sexually selected infanticide. Scientific Reports. 2017;7: 45222 10.1038/srep45222 28332613 PMC5362984

[14] Fattebert J, Balme GA, Robinson HS, Dickerson T, Slotow R, Hunter LTB. Population recovery highlights spatial organization dynamics in adult leopards. Journal of Zoology. 2016;299: 153–162. 10.1111/jzo.12344

[15] Shannon G, Slotow R, Durant SM, Sayialel KN, Poole J, Moss C, et al Effects of social disruption in elephants persist decades after culling. Frontiers in Zoology. 2013;10: 62 10.1186/1742-9994-10-62 24152378 PMC3874604

[16] Loveridge AJ, Searle AW, Murindagomo F, Macdonald DW. The impact of sport-hunting on the population dynamics of an African lion population in a protected area. Biological Conservation. 2007;134: 548–558. 10.1016/j.biocon.2006.09.010

[17] Ausband DE, Stansbury CR, Stenglein JL, Struthers JL, Waits LP. Recruitment in a social carnivore before and after harvest. Animal Conservation. 2015;18: 415–423. 10.1111/acv.12187

[18] Emslie RH, Milliken T, Talukdar B, Ellis S, Adcock K, Knight MH. African and Asian rhinoceroses–status, conservation and trade. A report from the IUCN Species Survival Commission (IUCN SSC) African and Asian Rhino Specialist Groups and TRAFFIC to the CITES Secretariat pursuant to Resolution Conf. 9.14 (Rev. CoP15). 2016.

[19] Ferreira SM, Greaver CC, Knight MH. Assessing the population performance of the black rhinoceros in Kruger National Park. South African Journal of Wildlife Research. 2011;41: 192–204. 10.3957/056.041.0206

[20] Hübschle AM. The social economy of rhino poaching: Of economic freedom fighters, professional hunters and marginalized local people. Current Sociology. 2017;65: 427–447. 10.1177/0011392116673210

[21] Moneron S, Okes N, Rademeyer J. Pendants, powder and pathways. Pretoria, South Africa: TRAFFIC, East/Southern Africa; 2017.

[22] Owen-Smith RN. Megaherbivores: The influence of very large body size on ecology. Cambridge, United Kingdom: Cambridge University Press; 1988.

[23] Linklater WL, Hutcheson IR. Black rhinoceros are slow to colonize a harvested neighbour’s range. African Journal of Wildlife Research. 2010;40: 58–63. 10.3957/056.040.0107

[24] Greaver C, Ferreira S, Slotow R. Density-dependent regulation of the critically endangered black rhinoceros population in Ithala Game Reserve, South Africa. Austral Ecology. 2014;39: 437–447. 10.1111/aec.12101

[25] Mating Goddard J. and courtship of the black rhinoceros. African Journal of Ecology. 1966;4: 69–75. 10.1111/j.1365-2028.1966.tb00883.x

[26] Garnier JN, Bruford MW, Goossens B. Mating system and reproductive skew in the black rhinoceros. Molecular Ecology. 2001;10: 2031–2041. 10.1046/j.0962-1083.2001.01338.x 11555246

[27] Adcock K. The relevance of “territorial” behaviour in black rhino to their population management. Proceedings of a Symposium on Rhinos as Game Ranch Animals. Onderstepoort, South Africa; 1994.

[28] Hitchins PM. Preliminary findings in a radio telemetric study on the black rhinoceros in Hluhluwe Game Reserve, Zululand. Proceedings of a symposium on Biotelemetry. Pretoria; 1971. pp. 79–100.

[29] Hitchins PM, Anderson JL. Reproduction, population, characteristics and management of the black rhinoceros Diceros bicornis minor in the Hluhluwe/ Corridor/Umfolozi Game Reserve Complex. South African Journal of Wildlife Research. 1983;13: 78–85.

[30] Schenkel R, Schenkel-Hulliger L. Ecology and behaviour of the black rhinoceros (Diceros Bicornis L.): a field study. Hamburg and Berlin, Paul Parey (Mammalia Depicta); 1969.

[31] Hall-Martin A. Black rhinoceros in Southern Africa. Oryx. 1979;15: 27–32. 10.1017/S0030605300016343

[32] Hall-Martin A, Knight MH. Conservation and management of black rhinoceros in South African national parks. Proceedings of a Symposium on Rhinos as Game Ranch Animals. Onderstepoort, South Africa; 1994.

[33] Gertenbach WPD. Landscapes of the Kruger National Park. Koedoe. 1983;26: 9–121. 10.4102/koedoe.v26i1.591

[34] Emslie RH, Adcock K, Hansen HB. Fine tuning rhino management group age class system. Rhino Management Group Report; 1995 pp. 1–17.

[35] R Core Team. R: A language and environment for statistical computing. Vienna, Austria: R Foundation for Statistical Computing; 2019 Available: https://www.R-project.org/

[36] Allee WC. Animal aggregations. Chicago: The University of Chicago Press; 1931.

[37] Courchamp F, Clutton-Brock T, Grenfell B. Inverse density dependence and the Allee effect. Trends in Ecology & Evolution. 1999;14: 405–410. 10481205 10.1016/s0169-5347(99)01683-3

[38] Wells H, Strauss EG, Rutter MA, Wells PH. Mate location, population growth and species extinction. Biological Conservation. 1998;86: 317–324. 10.1016/S0006-3207(98)00032-9

[39] Gascoigne J, Berec L, Gregory S, Courchamp F. Dangerously few liaisons: a review of mate-finding Allee effects. Population Ecology. 2009;51: 355–372. 10.1007/s10144-009-0146-4

[40] Clutton-Brock TH, Gaynor D, Kansky R, MacColl AD, McIlrath G, Chadwick P, et al Costs of cooperative behaviour in suricates (Suricata suricatta). Proceedings of the Royal Society B: Biological Sciences. 1998;265: 185–190. 10.1098/rspb.1998.0281 9493405 PMC1688874

[41] Lent PC, Fike B. Home ranges, movements and spatial relationships in an expanding population of black rhinoceros in the Great Fish River Reserve, South Africa: research article. South African Journal of Wildlife Research. 2003;33: 109–118.

[42] Hrabar H, du Toit J. Dynamics of a protected black rhino (Diceros bicornis) population: Pilanesberg National Park, South Africa. Animal Conservation. 2005;8: 259–267. 10.1017/S1367943005002234

[43] Tuyttens FAM, Delahay RJ, Macdonald DW, Cheeseman CL, Long B, Donnelly CA. Spatial perturbation caused by a badger (Meles meles) culling operation: implications for the function of territoriality and the control of bovine tuberculosis (Mycobacterium bovis). Journal of Animal Ecology. 2000;69: 815–828. 10.1046/j.1365-2656.2000.00437.x 29313991

[44] Balme GA, Slotow R, Hunter LTB. Impact of conservation interventions on the dynamics and persistence of a persecuted leopard (Panthera pardus) population. Biological Conservation. 2009;142: 2681–2690. 10.1016/j.biocon.2009.06.020

[45] Gosselin J, Zedrosser A, Swenson JE, Pelletier F. The relative importance of direct and indirect effects of hunting mortality on the population dynamics of brown bears. Proceedings of the Royal Society B: Biological Sciences. 2015;282: 20141840 10.1098/rspb.2014.1840 25392469 PMC4262167

[46] Goddard J. Home range, behaviour, and recruitment rates of two black rhinoceros populations. African Journal of Ecology. 1967;5: 133–150. 10.1111/j.1365-2028.1967.tb00768.x

[47] Plotz RD, Linklater WL. Black rhinoceros (Diceros bicornis) calf succumbs after lion predation attempt: implications for conservation management. African Zoology. 2009;44: 283–287. 10.3377/004.044.0216

[48] Brain C, Forge O, Erb P. Lion predation on black rhinoceros (Diceros bicornis) in Etosha National Park. African Journal of Ecology. 1999;37: 107–109. 10.1046/j.1365-2028.1999.00137.x

[49] Ahmad AH, Payne J, Zainuddin ZZ. Preventing the extinction of the Sumatran rhinoceros. Journal of Indonesian Natural History. 2013;1: 12.

