# Supplementary figures and images for: Age structure changes indicate direct and indirect population impacts in illegally harvested black rhino
Source: PLoS One. 2020 Jul 29;15(7):e0236790. doi: 10.1371/journal.pone.0236790 (PMC7390388; doi:10.1371/journal.pone.0236790)

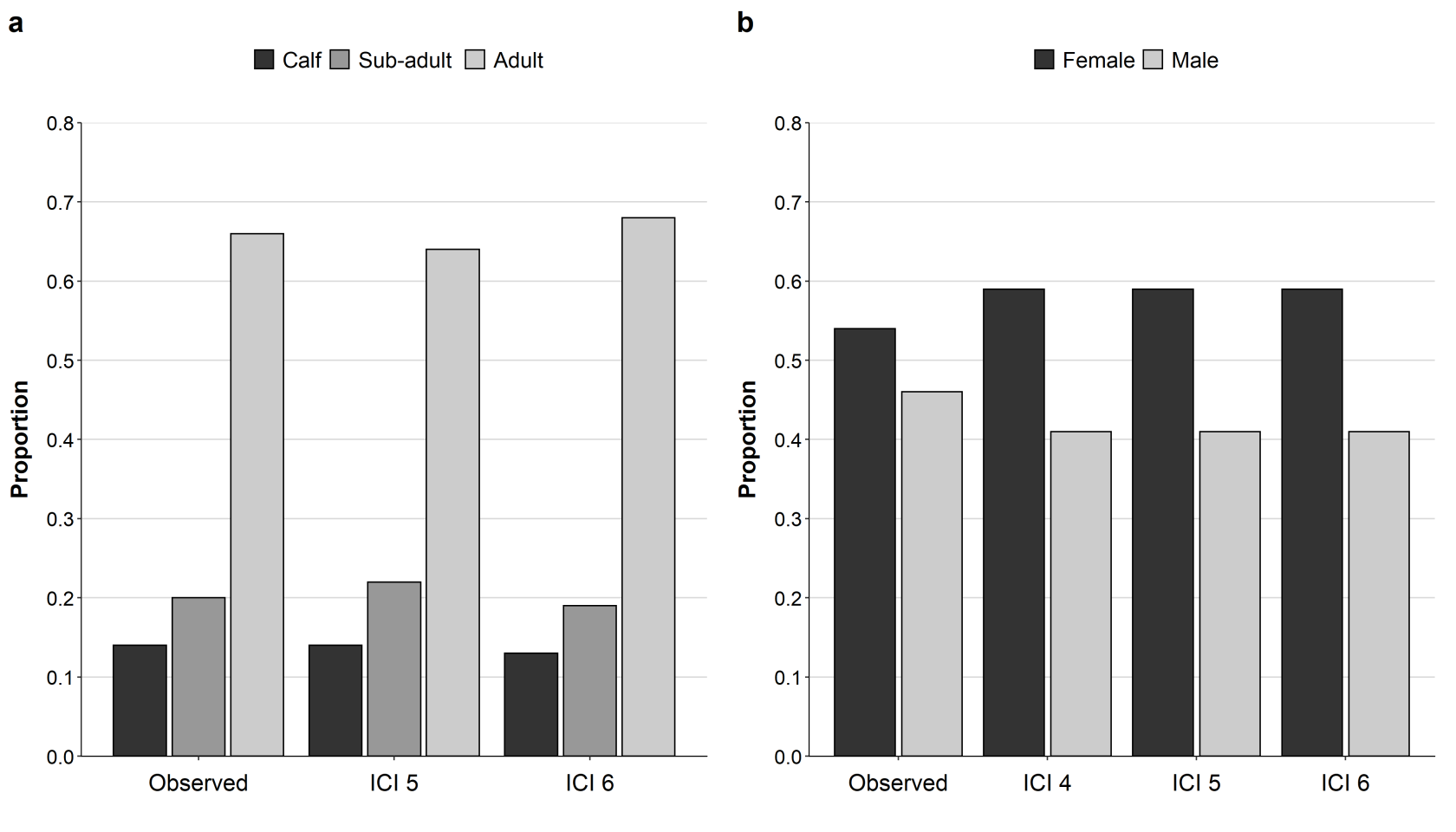

Supplement: S1 Fig — (a) Age group proportions of black rhino predicted in 2018 under ‘no sex/age bias + calves’ for ICI 5 and 6 compared to those observed in 2018. (b) Proportions of females and males predicted in 2018 under ‘no sex/age bias + calves’ for ICI 4, 5 and 6 compared to those observed in 2018. (TIF) [file pone.0236790.s001.tif]
