# Supplementary material for: Age structure changes indicate direct and indirect population impacts in illegally harvested black rhino
Source: PLoS One. 2020 Jul 29;15(7):e0236790. doi: 10.1371/journal.pone.0236790 (PMC7390388; doi:10.1371/journal.pone.0236790)
Supplement: S1 Table — Chi-squared statistics compare subsequent years to 2009. (DOCX) [file pone.0236790.s002.docx]

**S1 Table.** **Operational sex ratios (number of adult females per adult male) of black rhino observed in 2009 and 2013-2018.** Chi-squared statistics compare subsequent years to 2009.

| **Year** | **Operational sex ratio** | **χ^2^** | ***P* value** |
| --- | --- | --- | --- |
| 2009 | 1.152 | - | - |
| 2013 | 0.91 | 1.182 | 0.2769 |
| 2014 | 0.778 | 1.818 | 0.1741 |
| 2015 | 0.786 | 2.741 | 0.0978 |
| 2016 | 1.121 | 0.024 | 0.8781 |
| 2017 | 1.254 | 0.251 | 0.6161 |
| 2018 | 1.170 | 0.007 | 0.9329 |
