# Supplementary material for: Age structure changes indicate direct and indirect population impacts in illegally harvested black rhino
Source: PLoS One. 2020 Jul 29;15(7):e0236790. doi: 10.1371/journal.pone.0236790 (PMC7390388; doi:10.1371/journal.pone.0236790)
Supplement: S2 Table — Chi-squared statistics compare projected to observed 2018 proportions. (DOCX) [file pone.0236790.s003.docx]

**S2 Table.** **Projected black rhino age group proportions in 2018 under different poaching and fecundity scenarios.** Chi-squared statistics compare projected to observed 2018 frequencies.

| **Scenario** | **Calf** | **Sub-adult** | **Adult** | **χ^2^** | ***P* value** |
| --- | --- | --- | --- | --- | --- |
| *ICI = 3 years* |  |  |  |  |  |
| Recorded | 0.2 | 0.37 | 0.43 | 146.96 | <0.01 |
| No sex bias + calves | 0.18 | 0.32 | 0.5 | 56.76 | <0.01 |
| No sex/age bias + calves | 0.19 | 0.29 | 0.52 | 48.75 | <0.01 |
| *ICI = 4 years* |  |  |  |  |  |
| Recorded | 0.18 | 0.34 | 0.48 | 79.48 | <0.01 |
| No sex bias + calves | 0.16 | 0.30 | 0.55 | 22.79 | <0.01 |
| No sex/age bias + calves | 0.16 | 0.25 | 0.59 | 11.66 | <0.01 |
| *ICI = 5 years* |  |  |  |  |  |
| Recorded | 0.16 | 0.32 | 0.52 | 45.16 | <0.01 |
| No sex bias + calves | 0.13 | 0.29 | 0.58 | 17.17 | <0.01 |
| No sex/age bias + calves | 0.14 | 0.22 | 0.64 | 1.23 | 0.540 |
| *ICI = 6 years* |  |  |  |  |  |
| Recorded | 0.15 | 0.30 | 0.54 | 27.33 | <0.01 |
| No sex bias sex + calves | 0.12 | 0.27 | 0.61 | 8.64 | 0.013 |
| No sex/age bias + calves | 0.13 | 0.19 | 0.68 | 0.71 | 0.703 |
