# Supplementary material for: Age structure changes indicate direct and indirect population impacts in illegally harvested black rhino
Source: PLoS One. 2020 Jul 29;15(7):e0236790. doi: 10.1371/journal.pone.0236790 (PMC7390388; doi:10.1371/journal.pone.0236790)
Supplement: S3 Table — Chi-squared statistics compare projected to observed 2018 ratios. (DOCX) [file pone.0236790.s004.docx]

**S3 Table.** **Projected operational sex ratios (number of adult females per adult male) of black rhino in 2018 under different poaching and fecundity scenarios.** Chi-squared statistics compare projected to observed 2018 ratios.

| **Scenario** | **Operational sex ratio** | **χ^2^** | ***P* value** |
| --- | --- | --- | --- |
| *ICI = 3 years* |  |  |  |
| Recorded | 2.97 | 46.19 | <0.01 |
| No sex bias + calves | 1.94 | 14.37 | <0.01 |
| No sex/age bias + calves | 1.48 | 4.52 | 0.034 |
| *ICI = 4 years* |  |  |  |
| Recorded | 3.14 | 45.42 | <0.01 |
| No sex bias + calves | 1.96 | 13.48 | <0.01 |
| No sex/age bias + calves | 1.44 | 3.26 | 0.071 |
| *ICI = 5 years* |  |  |  |
| Recorded | 3.32 | 45.87 | <0.01 |
| No sex bias + calves | 1.97 | 12.57 | <0.01 |
| No sex/age bias + calves | 1.45 | 3.30 | 0.069 |
| *ICI = 6 years* |  |  |  |
| Recorded | 3.44 | 46.22 | <0.01 |
| No sex bias + calves | 1.96 | 11.89 | <0.01 |
| No sex/age bias + calves | 1.44 | 2.99 | 0.084 |
|  |  |  |  |
