# Supplementary material for: Age structure changes indicate direct and indirect population impacts in illegally harvested black rhino
Source: PLoS One. 2020 Jul 29;15(7):e0236790. doi: 10.1371/journal.pone.0236790 (PMC7390388; doi:10.1371/journal.pone.0236790)
Supplement: S4 Table — Chi-squared statistics compare projected to observed 2018 ratios. (DOCX) [file pone.0236790.s005.docx]

**S4 Table.** **Projected ratios of adult female black rhino per dependent calf in 2018 under different poaching and fecundity scenarios.** Chi-squared statistics compare projected to observed 2018 ratios.

| **Scenario** | **Adult females per calf** | **χ^2^** | ***P* value** |
| --- | --- | --- | --- |
| *ICI = 3 years* |  |  |  |
| Recorded | 1.59 | 17.18 | <0.01 |
| No sex bias + calves | 1.85 | 5.33 | 0.021 |
| No sex/age bias + calves | 1.67 | 12.17 | <0.01 |
| *ICI = 4 years* |  |  |  |
| Recorded | 2.00 | 3.57 | 0.059 |
| No sex bias + calves | 2.32 | 0. 30 | 0.586 |
| No sex/age bias + calves | 2.13 | 1.86 | 0.173 |
| *ICI = 5 years* |  |  |  |
| Recorded | 2.44 | 0.14 | 0.706 |
| No sex bias + calves | 2.86 | 0.44 | 0.509 |
| No sex/age bias + calves | 2.63 | 0.04 | 0.845 |
| *ICI = 6 years* |  |  |  |
| Recorded | 2.78 | 0.32 | 0.571 |
| No sex bias + calves | 3.33 | 1.91 | 0.167 |
| No sex/age bias + calves | 3.13 | 1.39 | 0.238 |
